# Antibiotic use in different hospital administrative categories: an overview of 10 years of a statewide surveillance program in Brazil

**DOI:** 10.1017/ash.2024.480

**Published:** 2025-01-13

**Authors:** Filipe Piastrelli, Denise Brandão de Assis, Geraldine Madalosso, Ícaro Boszczowski

**Affiliations:** 1 Infection Control Department, Hospital Alemão Oswaldo Cruz, São Paulo, Brazil; 2 Divisao de Infeccoes Hospitalares, Centro de Vigilancia Epidemiologica “Prof. Alexandre Vranjac” Centro de Controle de Doencas, Secretaria de Estado da Saude, São Paulo, SP, BR, Brazil; 3 Infection Control Department, Hospital Alemão Oswaldo Cruz and Infection Control Department Hospital das Clínicas, São Paulo, Brazil

## Abstract

**Objective:**

The present study aimed to describe ICU antibiotic use based on data reported from 2009 to 2018 to the Nosocomial Surveillance System (NSS) of the State Health Department in the State of Sao Paulo, Brazil.

**Design:**

Ecological study.

**Setting:**

Data obtained from hospitals located in the state of São Paulo, Brazil from 2009 to 2018.

**Participants:**

Intensive care units located at participant hospitals.

**Methods:**

Data on healthcare-associated infections, antibiotic usage, and bacterial identification were collected and reported monthly by hospitals. Antibiotic consumption was quantified as defined daily doses (DDD) per 1000 patient-days. The relationship between antibiotic use and bacterial resistance, categorized by hospital type and ICU complexity, was analyzed using statistical methods to assess correlations and significance.

**Results:**

Our findings reveal an escalating trend in antibiotic consumption over the study period, with a notable increase from 588.16 DDD per 1000 patient-days in the initial year to 943.12 DDD/1000 patient-days in the final year (p < 0.01). Cephalosporins emerged as the most frequently utilized class, accounting for 33.9% of total antibiotic consumption. Public hospitals exhibited significantly higher antibiotic use compared to private and philanthropic institutions, with a mean of 889.11 DDD/1000 patient-days in public hospitals compared to 849.07 DDD/1000 patient-days in private hospitals and 785.12 DDD/1000 patient-days in philanthropic hospitals (p < 0.05).

**Conclusions:**

The study provides critical insights into antibiotic use and resistance in different hospital settings, emphasizing the importance of tailored antimicrobial stewardship strategies.

## Introduction

Antimicrobial resistance (AMR) is a global public health threat that reduces drug effectiveness and undermines efforts to prevent and treat infectious diseases. The emergence of resistance is closely linked to the overuse of antimicrobials across various sectors, including human health, animal husbandry, and agriculture.^1^

In human health, the overprescription of antibiotics emerges as a notable risk factor driving the escalation of antibiotic resistance, albeit establishing a direct causal effect can be challenging. Consequently, infections caused by resistant microorganisms are associated with prolonged hospitalizations, escalated healthcare expenditures, and elevated mortality rates.^2,3^ Despite hospitals’ antibiotic consumption representing a fraction of global usage, the intensive care unit (ICU) serves as a critical focal point for resistance emergence and dissemination due to the frequent and intense antibiotic usage.^4^

Addressing AMR within the integrated framework of One Health stands as a paramount objective for the World Health Organization (WHO), as articulated in the Global Action Plan on Antimicrobial Resistance. The aim is to fortify monitoring and surveillance endeavors regarding antimicrobial utilization and AMR.^1^

A comprehensive understanding of antibiotic utilization is imperative for formulating effective interventions in antibiotic stewardship. However, surveillance in this context is intricate, with heterogeneous patterns of antibiotic usage observed even within the same country across different healthcare facilities.^5–7^

Brazil’s healthcare system is characterized by a complex structure featuring distinct financing models based on administrative categories: public, private, and philanthropic. Public hospitals can be government-administered or privately operated with public funding. Philanthropic hospitals operate as private entities but are nonprofit, while private hospitals operate for profit.^8^ These administrative nuances give rise to varying resource allocations and organizational structures, yet data elucidating disparities in antibiotic utilization are scarce.

Establishing a well-structured surveillance system is pivotal for comprehending antibiotic utilization and bacterial resistance patterns. Surveillance programs have been instituted to monitor antibiotic usage and resistance at various echelons. In Brazil, a national surveillance system has been monitoring antibiotic utilization in ICUs since 2014.^9^ Moreover, in 2017, Brazil entered the Global Antimicrobial Resistance Surveillance System (GLASS) and initiated the National Surveillance Program on Antimicrobial Resistance (BR-GLASS).^10^

Over a decade ago, in 2004, a regional center for healthcare infection surveillance was established in Sao Paulo, Brazil’s most populous state. This initiative has substantially enhanced the monitoring of healthcare-associated infections (HAIs), AMR, and antibiotic utilization in ICUs. Initially, 457 hospitals reported data to the system, a figure that has since burgeoned to 724 institutions by 2018.^11,12^

The present study aimed to describe ICU antibiotic use based on data reported to the Nosocomial Surveillance System (NSS) of the State Health Department in the State of Sao Paulo.

## Material and methods

### Study design and data collection

This ecological study describes antibiotic utilization at an aggregated level in adult intensive care units reported to São Paulo SHD (State Health Department) from 2009 to 2018.

At the onset of each calendar year, hospitals across the State of São Paulo receive an Excel spreadsheet encompassing monthly data on HAIs, antibiotic usage, and bacterial identification from blood cultures. The nosocomial infection control department of each hospital is responsible for reporting the number of primary bloodstream infections (BSIs), bacterial identification, and resistance pattern, and the quantity of antimicrobial vials utilized in the ICU. The spreadsheet automatically converts the number of filled vials into defined daily doses (DDD) as stipulated by the WHO.^13^ Completed spreadsheets are transmitted monthly to the State Health Department.

We included hospitals that reported a minimum of 500 patient-days each year. Data were pooled and analyzed as described below.

### Antibiotic use

Hospitals reported the utilization of the following antimicrobial agents: ampicillin-sulbactam, ciprofloxacin, moxifloxacin, levofloxacin, ceftriaxone, ceftazidime, cefotaxime, cefepime, piperacillin-tazobactam, ertapenem, imipenem, meropenem, polymyxin B, colistin, linezolid, vancomycin, and teicoplanin. Antibiotics were categorized into classes, and the data were presented as DDD per 1000 patient-days.

### Intensive care units

ICUs were categorized according to hospital administrative classification (private, public, and philanthropic) and complexity. An ICU was designated as high complexity when the average mechanical ventilation rate (%MV) exceeded 50%, and low complexity when the average mechanical ventilation rate was below 50%.

Public hospitals were further subdivided into two administrative subcategories: Social Health Organization (SHO), which operates with private administration supplemented by governmental resources, and direct public administration (DPA).

### Bacterial resistance and antibiotic use

Data regarding multidrug-resistant organisms (MDRO) isolated from blood cultures of ICU patients were reported in two formats throughout the study period: (1) from 2009 to 2011, both primary and secondary BSIs were documented; (2) from 2012 to 2018, only primary BSI cases were recorded. Primary and secondary BSIs adhere to the CDC criteria for central line-associated bloodstream infection (CLABSI) surveillance. Primary BSI is defined as “a laboratory-confirmed bloodstream infection not secondary to an infection at another body site,” while secondary BSI is described as “a bloodstream infection believed to have originated from a site-specific infection at another body site”.^14^

MDRO were categorized based on phenotypical characteristics, including methicillin-resistant *Staphylococcus aureus* (MRSA), third-generation cephalosporin (3GC)-resistant *Enterobacterales*, and carbapenem-resistant gram-negative bacilli (CR-GNB). Subsequently, the incidence and proportion of each MDRO group were calculated.

Incidence of resistance phenotypes and correlation with antibiotic usage were determined for MRSA and glycopeptide use, 3GC-resistant *Enterobacterales* and carbapenem use, and CR-GNB and polymyxin use.

### Statistical analyses

The overall pooled mean was calculated for each therapeutic class across hospitals and subgroups. Given the non-normal data distribution, the Kruskal–Wallis test was used for group comparisons, with Mann–Whitney plus Bonferroni correction for pairwise comparisons. Spearman’s coefficient assessed antibiotic utilization-resistance correlation.

Data analysis was performed with IBM-SPSS for Windows software version 25.0. A *P*-value <0.05 was considered statistically significant.

## Results

### Setting and antibiotic use

The average number of hospitals reporting annual data on antibiotic use in the study period was 386 (332–420) per year, with private hospitals being the most frequent administrative category (46.7%) (Table 1).

**Table 1.** Number of hospitals reporting to the epidemiological surveillance system in the state of São Paulo, 2009–2018

	2009	2010	2011	2012	2013	2014	2015	2016	2017	2018
Number of hospitals	330	355	368	377	384	398	411	406	413	420
Patient-day	1334135	1456582	1556547	1636265	1755132	1840916	1921900	1983981	2005019	2000489
Philanthropic	92	94	102	104	104	105	108	106	105	107
Private	148	161	167	173	182	189	194	191	201	199
Public	92	100	99	100	98	104	109	109	109	114
SHO	19	21	23	25	26	26	26	26	27	27
DPA	73	70	76	75	72	78	83	83	82	87

SHO, Social Health Organization; DPA, Direct Public Administration.

**Figure 1.**
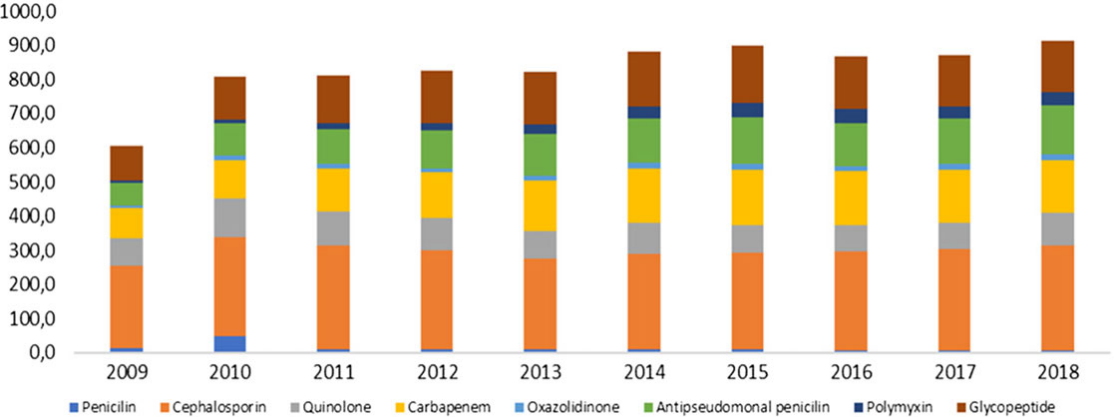
Antimicrobial use by therapeutic class between 2009 and 2018 in DDD/1000 patient-days.

Total antibiotic consumption in ICUs escalated from 588.16 DDD per 1000 patient-days in the initial year to 943.12 DDD/1000 patient-days in the final year (*P* < 0.01). Cephalosporins constituted the most frequently utilized antibiotic class (33.9%), with no significant variance observed over the study period. Following cephalosporins, glycopeptides (147.52 DDD/1000 patient-days) and carbapenems (140.76 DDD/1000 patient-days) ranked as the second and third most utilized groups, respectively (Figure 1).

The DDD of antibiotics was higher in public hospitals (mean 889.11 DDD/1000 patients-day) than in private hospitals (mean 849.07 DDD/1000 patients-day) and philanthropic hospitals (mean 785.12 DDD/1000 patients-day) (*P* < 0.05), but there is no significant difference between private and philanthropic hospitals. Use in philanthropic institutions was significantly higher for cephalosporins and quinolones. Private hospitals presented greater use for linezolid, while public facilities showed higher use for carbapenems, polymyxins, and glycopeptides (Table 2).

**Table 2.** Antibiotic use by therapeutic class by administrative type in DDD/1000 patients-day

Therapeutic class	Administrative type			
	Philanthropic	Private	Public	p
Penicillin	8.52 (1.25)	15.57 (0.56)	9.20 (1.20)	NS
Cephalosporin	308.48 (263.95)	280.69 (220.68)	272.38 (203.97)	<0.001^a^
Quinolone	101.96 (59.38)	89.50 (48.83)	72.34 (32.66)	<0.001^a^
Carbapenem	120.22 (99.05)	135.65 (114.23)	170.09 (158.01)	<0.001^a^
Oxazolidinone	4.07 (0.0)	24.54 (0.0)	8.10 (0.0)	<0.001^a^
Antipseudomonal penicillin	95.83 (84.31)	128.34 (118.87)	125.03 (112.03)	<0.001^b^
Polymyxin	22.01 (3.13)	22.29 (8.28)	46.69 (24.57)	<0.001^a^
Glycopeptide	120.25 (98.64)	143.15 (119.91)	182.23 (167.40)	<0.001^a^
Total	785.12 (744.30)	849.07 (730.57)	889.11 (796.01)	<0.001^c^

DDD, defined daily dose. a, Significant difference between all groups. b, No difference between groups private and public. c, No difference between groups philanthropic and private.

In public hospitals, total antibiotic use in the SHO subcategory was higher than in DPA (928.84 and 871.67 DDD/1000 patients-day, respectively – *P* < 0.001). Additionally, SHO exhibited higher consumption across all antibiotic classes except for cephalosporins and quinolones (Table 3).

**Table 3.** Antibiotic use in public hospitals by subgroup between 2009 and 2018 in DDD/1000 patients-day

Therapeutic class	Administration type in public hospitals		
	SHO	DPA	p
Penicillin	10.67 (2.20)	8.72 (0.87)	**<0.001**
Cephalosporin	231.64 (212.59)	285.10 (201.79)	>0.05
Quinolone	67.70 (34.41)	73.79 (32.32)	>0.05
Carbapenem	208.01 (185.72)	158.25 (148.05)	**<0.001**
Oxazolidinone	9.83 (0.0)	7.57 (0.0)	>0.05
Antipseudomonal penicillin	131.15 (127.43)	123.03 (108.12)	**<0.05**
Polymyxin	74.47 (56.04)	38.02 (17.88)	**<0.001**
Glycopeptide	202.46 (189.85)	175.91 (155.38)	**<0.001**
Total	928.84 (883.61)	871.67 (765.26)	**<0.001**

SHO, Social Health Organization – private administration; DPA, direct public administration.

ICUs classified as high-complexity demonstrated greater utilization of carbapenems, polymyxins, and glycopeptides compared to lower complexity ICUs (Table 4).

**Table 4.** Antibiotic use according to ICU complexity from 2009 to 2018 in DDD/1000 patient-days

	ICU complexity		
Therapeutic class	Low	High	p
Penicillin	12.02 (0.41)	11.90 (2.96)	<0.001
Cephalosporin	276.70 (225.51)	309.36 (222.79)	p = 0.802
Quinolone	85.07 (45.74)	96.32 (46.69)	p = 0.164
Carbapenem	130.40 (112.41)	167.41 (142.08)	<0.001
Oxazolidinone	16.76 (0.0)	9.44 (0.0)	<0.001
Antipseudomonal penicillin	116.10 (108.36)	125.70 (112.83)	p = 0.183
Polymyxin	24.06 (7.81)	40.79 (16.28)	<0.001
Glycopeptide	135.34 (117.92)	178.83 (152.08)	<0.001
Total	802.52 (726.26)	946.32 (814.89)	<0.001

ICU, intensive care unit.

### Correlation between antibiotic use and bacterial resistance

**Figure 2.**
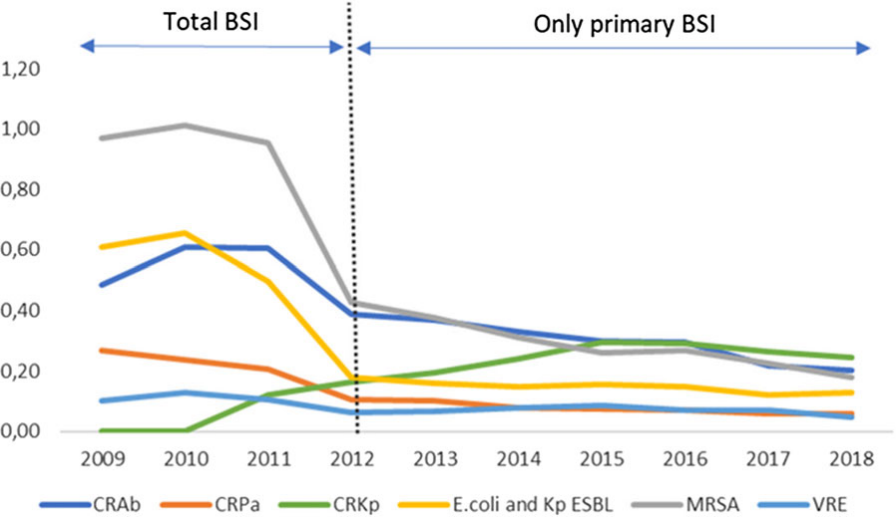
Incidence of resistant bacteria by phenotypic profile of resistance in the period 2009 to 2018. BSI, bloodstream infection; CRAb, carbapenem-resistant *Acinetobacter baumannii*; CRPa, carbapenem-resistant *Pseudomonas aeruginosa*; CRKp, carbapenem-resistant *Klebsiella pneumoniae*; ESBL, extended-spectrum beta-lactamase; MRSA, methicillin-resistant *S.aureus*; VRE, vancomycin-resistant *Enterococcus sp*.

The incidence of all resistance phenotypes, except vancomycin-resistant *Enterococcus sp.* (VRE), showed significant variation between 2009 and 2018 because of the difference in the report method adopted in two periods: 2009–2011 and 2012–2018. The incidence of carbapenem-resistant *Acinetobacter baumannii* (CRAb) and MRSA decreased between 2012 and 2018 when only primary BSI was reported (*P* < 0.05). However, in this period, the proportion of CRAb increased significantly (*P* < 0.001). The incidence and proportion of carbapenem-resistant *Klebsiella pneumoniae* (CRKp) showed a substantial increase between 2012 and 2018 (Figure 2).

The proportion of MDRO was higher in public hospitals compared to private and philanthropic institutions (*P* <0.05). However, only CRAb exhibited no difference when comparing public and private hospitals (Table 5). For public hospitals, MRSA prevalence was higher in SHO subgroup than in the DPA subgroup (*P* <0.05). No disparity was observed in the proportion of the other patterns assessed.

Positive correlations were identified between glycopeptide use and MRSA incidence, as well as polymyxin use and carbapenem-resistant gram-negative bacilli (CR-GNB) incidence. Additionally, a weak negative correlation was observed between carbapenem use and third-generation cephalosporin (3GC)-resistant *Enterobacterales* incidence (Table 6).

**Table 5.** Proportion of resistant bacteria by phenotypic profile of resistance and administration type in the period 2009 to 2018

Administration type	CRAb	CRPa	CRKp	ESBL	MRSA	VRE
Philanthropic	65,6%	28,2%	26,3%	24,6%	62,9%	23,0%
Private	71,4%*	34,1%	29,6%	23,8%	56,9%	22,4%
Public	76,8%*	44,0%*	37,0%*	29,3%*	72,7%*	40,7%*

CRAb, carbapenem-resistant *Acinetobacter baumannii*; CRPa, carbapenem-resistant *Pseudomonas aeruginosa*; CRKp, carbapenem-resistant *Klebsiella pneumoniae*; ESBL, extended-spectrum beta-lactamase; MRSA, methicillin-resistant *S.aureus*; VRE, vancomycin-resistant *Enterococcus sp*.

**Table 6.** Spearman’s correlation coefficient for antibiotic use and bacterial resistance incidence between 2009 a 2018

Glycopeptides and MRSA	Carbapenems and 3GC-resistant *Enterobacterales*	Polymyxins and CR-GNB
r = 0.140 (**p < 0.01**)	r = –0.076 (**p < 0.01**)	r = 0.406 *(***p < 0.01***)*

MRSA, methicillin-resistant *Staphylococcus aureus*; 3GC, third-generation cephalosporin-resistant *Enterobacterales*; CR-GNB, carbapenem-resistant gram-negative bacilli.

## Discussion

Improving the use of antibiotics and the knowledge of bacterial resistance represent key objectives of the Global Action Plan on Antimicrobial Resistance, as presented by the WHO and endorsed in Brazil.^1,15^ A significant strategy involves investigating the relationship between antimicrobial use and resistance. This study aimed to explore the patterns of antibiotic utilization in the ICUs and their correlation with bacterial resistance in bloodstream isolates within the Health Surveillance System of a Brazilian state. Furthermore, we examined antibiotic use across different groups of hospitals categorized by administrative type – public, private, and philanthropic – a unique feature of Brazil’s healthcare sector.

Until 2015, antibiotic use data at the hospital level based on a systematic national surveillance system were restricted to a few countries,^7,16–18^ while antibiotic use reports based on regional statistics^5,17,19^ or restricted to local intensive care units^20–22^ were relatively more common. However, the establishment and expansion of GLASS have significantly increased surveillance of AMR and antibiotic use with national data from different regions of the globe. In 2021, 109 countries enrolled in GLASS.^23^ Our study presents BSIs data from an AMR surveillance period before BR-GLASS, describing antibiotic use in intensive care units in the State of São Paulo, whose SHD was a national pioneer in the composition of an information surveillance system for hospital infections.

The global use of antibiotics in the ICU found in our study is lower than previously reported in ICU by other studies: 916 DDD/1000 patients-day in Porto Alegre, Brazil, 1140 DDD/1000 patients-day in Germany, 1143 DDD/1000 patients-day in Switzerland, 1466 DDD/1000 patients-day in France and 1471 DDD/1000 patients-day in Catalonia, Spain.^3,5,7,16,21^ However, different drug surveillance methods may explain multinational antibiotic use discrepancies.

In our study, cephalosporins were the most used therapeutic class, which differs from the practice observed in European ICUs in which penicillins, especially using amoxicillin-clavulanate, represent the group of antibiotics most used in intensive care units.^5,7,16,24^ A Brazilian point-prevalence study showed that ceftriaxone was the most prescribed antibiotic in the sample evaluated, mainly for treating respiratory and urinary infections.^25^ The reasons for these differences in prescription patterns, favoring either amoxicillin-clavulanate or ceftriaxone as the first choice, remain unclear and warrant further investigation, possibly involving cultural factors or cost considerations.

Otherwise, cephalosporins use exhibited a decreasing trend over the observed period, possibly associated with an increase in the proportion of gram-negative bacteria resistant to these drugs. Globally, there is a trend toward increasing MDRO gram-negative in intensive care units,^26–28^ which is generally associated with an increase in hospital length of stay, mortality, and hospital costs.^27^ Our study also observed an increase in the proportion of CRAb and CRKp over the 10-year period.

Few studies have evaluated the difference in antibiotic use by administrative type. A French study,^16^ showed a higher antibiotic use in private hospitals than in public institutions, a trend not observed in our study. Public hospitals exhibited the highest antibiotic use, while private institutions used more antibiotics than philanthropic hospitals. A previous Brazilian survey on community and hospital antibiotic use observed a greater burden of resistant bacteria in public hospitals, possibly attributable to patient overload in these institutions, which may affect healthcare quality.^29^

Moreover, among public hospitals, we observed higher use of carbapenems, glycopeptides, and polymyxins in SHO hospitals. Notably, there was a positive correlation between glycopeptide use and MRSA incidence in SHO hospitals but not in DPA hospitals. For both types of administration, there was a positive correlation between polymyxins and the incidence of CR-GNB and no correlation between the use of meropenem and the incidence of 3GC-resistant *Enterobacterales*. Because these are unique administration profiles in other countries, there are no comparative data for these subgroups. SHO represents the private administration of public institutions with governmental funding.^30,31^ The correlation of antibiotic use with bacterial resistance incidence suggests more efficient resource utilization, which has significant implications for Brazilian public health policy. However, further studies are needed to evaluate this hypothesis.

Typically, comparisons of antibiotic utilization among ICU types are based on the specialty of care, such as cardiology, medical and surgical, and there are limited studies describing differences based on the level of care complexity.^32^ In our study, ICUs were stratified into high or low complexity based on the rate of mechanical ventilation. Although it is not a formally validated parameter for the definition of care complexity, the percentage of mechanical ventilation usage allows inference of patient severity in the unit and has been described in another study as a method of ICU stratification for data benchmarking.^33^

We observed higher antibiotic utilization and incidence of MRSA, carbapenem-resistant gram-negative bacteria (CR-GNB), and third-generation cephalosporin (3GC)-resistant *Enterobacterales* in high-complexity ICUs. C Critically ill patients in the ICU, along with the associated risks of delayed therapy, contribute to a lower threshold for initiating antimicrobial treatment, and this hypothesis helps to explain the observed result.

We found a weak negative correlation between the proportion of 3GC-resistant *Enterobacterales* and carbapenems use. His finding was unexpected; however, one possible explanation is the concurrent administration of carbapenems in the treatment of CR-GNB,, which may be associated with the observed negative correlation between the proportion of 3GC-resistant *Enterobacterales* and CR-GNB.

Our study has several limitations. First, the ecological study design cannot establish the cause-and-effect relationship between antibiotic use and resistance. Second, during the period observed, there was a change in the notification criteria of bacteremias, and this does not allow for continuous analysis of the resistance incidence over the entire period. Third, although a significant difference in antibiotic use was found based on the complexity of the ICU, the criteria employed for stratification require validation by other studies. Fourth, significant differences in antibiotic use and MDRO incidence were observed in the different hospital administrative categories. However, the study design does not allow us to establish the causes related to these findings.

In summary, we found substantial variations in antibiotic utilization and MDRO incidence across different hospital administrative categories, highlighting the importance of tailored interventions based on healthcare facility characteristics. Notably, we observed a higher proportion of MDRO in public hospitals compared to private and philanthropic institutions, suggesting potential areas for targeted interventions to mitigate resistance emergence. Furthermore, our findings revealed an unexpected negative correlation between the proportion of third-generation cephalosporin (3GC)-resistant *Enterobacterales* and carbapenem use, underscoring the complexity of AMR dynamics in healthcare settings. Despite these insights, our study has inherent limitations, including its ecological design and the need for validation of ICU complexity stratification criteria. Future research efforts should focus on elucidating the causal relationships between antibiotic use and resistance patterns, thereby facilitating the development of evidence-based antimicrobial stewardship strategies tailored to diverse healthcare contexts.
